# Contactless Remote Monitoring for the Detection of Drug Effects in People Living With Dementia: A Case Study on the Use of Mirtazapine to Treat Insomnia

**DOI:** 10.1192/bjo.2025.10750

**Published:** 2025-06-20

**Authors:** Ana Mirza-Davies, Eyal Soreq, David Sharp

**Affiliations:** Imperial College CR&T Dementia Research Institute, London, United Kingdom

## Abstract

**Aims:** Safe and effective prescribing in people living with dementia (PLWD) is particularly challenging due to the increased risk of adverse events, polypharmacy and potentially inappropriate medications. Cognitive impairment and reliance on caregivers to report symptoms can further complicate the assessment of drug benefits. This case study demonstrates how novel contactless monitoring could address these challenges by enabling remote evaluation of drug effects in PLWD.

**Methods:** We present the case of a 77-year-old gentleman with late onset Alzheimer’s disease enrolled on the CR&T MINDER cohort study and continuously monitored using the Withing’s Sleep Analyzer. He visited his GP with complaints of insomnia and was subsequently prescribed 15 mg of mirtazapine. He reported immediate beneficial effects, although noted that the drug made him drowsy. We evaluated his sleep by comparing baseline sleep metrics (a 2-week average 1 month before drug administration) with average sleep metrics 2 weeks after starting mirtazapine. Statistical analysis was performed using a paired t-test and a rolling average to assess trends over time.

**Results:** Calculated rolling average showed reductions in time spent awake overnight and in light sleep, while deep sleep and total sleep time increased. These trends were confirmed by period comparisons. Baseline deep sleep duration (M = 1.19 hrs, SD = 1.02 hrs), and total sleep time (M = 7.16 hrs, SD = 1.03 hrs) significantly increased 2 weeks post mirtazapine (deep sleep: M = 2.63 hrs, SD = 1.03 hrs); total sleep time: M = 7.87 hrs, SD = 0.47 hrs), t(13) = −3.639, p = 0.003, and t(13) = −2.256, p = 0.042. There was also a significant reduction in time spent awake during the night from baseline (M = 1.17 hrs, SD = 0.51 hrs) to 2 weeks post mirtazapine (M = 0.65 hrs, SD = 0.43 hrs), t(13) = 2.616, p = 0.0214.

**Conclusion:** This case study shows that contactless remote monitoring could be used objectively to evaluate the effects of mirtazapine on sleep in PLWD. Our results demonstrate that improvements in sleep detected by monitoring align with the participants’ reported benefits. These findings suggests continuous remote monitoring could provide valuable, timely insights into drug effects in PLWD, improving clinical decision-making and personalising care.

